# Neurovascular Development in *Pten* and *Tsc2* Mouse Mutants

**DOI:** 10.1523/ENEURO.0340-22.2023

**Published:** 2023-02-22

**Authors:** Mary Dusing, Candi L. LaSarge, Angela White, Lilian G. Jerow, Christina Gross, Steve C. Danzer

**Affiliations:** 1 Department of Anesthesia, Cincinnati Children’s Hospital Medical Center, Cincinnati, OH 45229; 2 Division of Neurology, Cincinnati Children’s Hospital Medical Center, Cincinnati, OH 45229; 3 Departments of Anesthesia and Pediatrics, University of Cincinnati, Cincinnati, OH 45229; 4 Center for Pediatric Neuroscience, Cincinnati Children’s Hospital, Cincinnati, OH 45229; 5 Neuroscience Graduate Program, University of Cincinnati, Cincinnati, OH 45219

**Keywords:** angiogenesis, focal cortical dysplasia, mTOR, mtoropathy, tuberous sclerosis, Vegf

## Abstract

Hyperactivation of the mechanistic target of rapamycin (mTOR) signaling pathway is linked to more than a dozen neurologic diseases, causing a range of pathologies, including excess neuronal growth, disrupted neuronal migration, cortical dysplasia, epilepsy and autism. The mTOR pathway also regulates angiogenesis. For the present study, therefore, we queried whether loss of *Pten* or *Tsc2*, both mTOR negative regulators, alters brain vasculature in three mouse models: one with *Pten* loss restricted to hippocampal dentate granule cells [DGC-*Pten* knock-outs (KOs)], a second with widespread *Pten* loss from excitatory forebrain neurons (FB-*Pten* KOs) and a third with focal loss of *Tsc2* from cortical excitatory neurons (f-*Tsc2* KOs). Total hippocampal vessel length and volume per dentate gyrus were dramatically increased in DGC-*Pten* knock-outs. DGC-*Pten* knock-outs had larger dentate gyri overall, however, and when normalized to these larger structures, vessel density was preserved. In addition, tests of blood-brain barrier integrity did not reveal increased permeability. FB-*Pten* KOs recapitulated the findings in the more restricted DGC-*Pten* KOs, with increased vessel area, but preserved vessel density. FB-*Pten* KOs did, however, exhibit elevated levels of the angiogenic factor VegfA. In contrast to findings with *Pten*, focal loss of *Tsc2* from cortical excitatory neurons produced a localized increase in vessel density. Together, these studies demonstrate that hypervascularization is not a consistent feature of mTOR hyperactivation models and suggest that loss of different mTOR pathway regulatory genes exert distinct effects on angiogenesis.

## Significance Statement

Here, we examined three mouse models to determine whether mechanistic target of rapamycin (mTOR) hyperactivation consistently drives brain hypervascularization. Both focal loss of *Pten* from dentate granule cells, and widespread loss from forebrain produced larger brain structures and corresponding increases in vascular growth, but normal vessel density. By contrast, focal cortical *Tsc2* lesions exhibited significantly increased vessel density. Findings indicate that hypervascularization is not characteristic of all mTOR hyperactivation models and suggest vascular changes may be driven by gene-specific effects.

## Introduction

Mutations in more than a dozen genes that regulate the mechanistic target of rapamycin (mTOR) pathway cause a variety of syndromes collectively known as mTORopathies (Crino, 2020). mTORopathies are characterized by brain structural defects, benign tumors, intellectual disability, autism and epilepsy (Mirzaa et al., 2016; Marsan and Baulac, 2018). Affected genes include *mTOR* itself, as well as negative regulators like tuberous sclerosis complex subunits 1 and 2 (*TSC1/2*) and phosphatase and tensin homolog (*PTEN*). Mutations in animal models directly increase mTOR signaling in affected neurons, leading to morphologic and physiological changes that include neuronal hypertrophy, dendritic and axonal sprouting and increased excitability (Zeng et al., 2009; Barrows et al., 2017; Switon et al., 2017; Skelton et al., 2020; Narvaiz et al., 2022).

Mutations in mTOR pathway genes can also lead to blood vessel abnormalities. In human cancers, where *mTOR* mutations are common, mutations contribute to tumor-induced angiogenesis (Wen et al., 2001; Tian et al., 2010; Karar and Maity, 2011). Increased vessel density has been described in focal cortical dysplasia (Wintermark et al., 2013), a condition commonly associated with *mTOR* mutations. Vascular abnormalities have also been observed in patients with tuberous sclerosis complex (Mühlebner et al., 2016; Chihi et al., 2019 Sun et al., 2021) and in animal models of the disease (Zhang et al., 2019; Kútna et al., 2020). Vascular abnormalities could be driven by several mechanisms. First, excess growth of neurons with mTOR pathway mutations can lead to enlarged brain structures, necessitating additional angiogenesis to support the larger structures. Second, mTOR pathway mutations commonly cause seizures that can disrupt the blood-brain barrier and induce vascular changes (van Vliet et al., 2012; Mendes et al., 2019). Finally, increased expression of genes involved in vascular remodeling has been observed in *mTOR* mutants and following pharmacological manipulation of mTOR signaling. Changes have been observed at the mRNA and protein levels in a variety of tissue types, including peripheral tumors and brain. Affected genes include vascular endothelial growth factor (*Vegf*), angiopoietin 1 and 2 (*Ang-1*, *Ang-2*), hypoxic inducible factor 1 (*Hif-1alpha*) and matrix metalloproteinases (Land and Tee, 2007; Parker et al., 2011; Feliciano et al., 2013; Xue et al., 2018; Broekaart et al., 2020).

Vascular abnormalities could be important disease-driving components for patients with *mTOR* mutations. Increased vascularity would provide better access to oxygen and nutrients for dysplastic neurons, facilitating aberrant growth akin to that observed in tumors. Vessel remodeling can also be associated with increased blood-brain barrier permeability. Increased permeability can initiate cascades of negative sequelae, including disrupted ion homeostasis, inflammatory effects of toxic serum proteins like albumin and seizures (Frigerio et al., 2012; Gorter et al., 2019).

Here, we queried whether loss of the mTOR pathway inhibitors *Pten* or *Tsc2* would also produce vascular changes in the brain. Three genetic models were used: the 1st targeting hippocampal granule cell progenitors [DGC-*Pten* knock-out (KO)], the 2nd targeting excitatory forebrain neurons (FB-*Pten* KO) and the 3rd producing focal *Tsc2* loss from cortical excitatory neurons. Neurons in both *Pten* models exhibit physiologic and morphologic abnormalities, while the animals exhibit spontaneous seizures (McMahon et al., 2012; Sperow et al., 2012; LaSarge et al., 2015, 2016, 2019; Santos et al., 2017; Arafa et al., 2019; Chen et al., 2019; White et al., 2020). These studies will help to establish whether vascular abnormalities are characteristic of mTORopathies.

## Materials and Methods

### Animals

All animal procedures were conducted in accordance with National Institutes of Health (NIH) and Institutional Animal Care and Use Committee (IACUC) guidelines. Gli1-CreER^T2^ (RRID:IMSR_JAX:007913), CamK2α-Cre (RRID:IMSR_JAX:005359), *Pten*^fl/fl^ (RRID:IMSR_JAX:006440), TdTomato^fl/fl^ (TdTom; RRID:IMSR_JAX:007914), and *Tsc2^fl/fl^* (RRID:IMSR_JAX:027458) mice were obtained from the Jackson laboratory and maintained as colonies in our vivarium. All animals were maintained on a C57BL/6 background, including the *Tsc2^fl/fl^* mice that were obtained as B6129SF2/J and subsequently crossed with C57BL/6 for eight generations. Littermates were distributed across all time points and groups where possible. The ARRIVE guidelines were followed for study design and reporting (Kilkenny et al., 2010).

### Labeling of vascular structure in DGC-*Pten* KOs

To examine the impact of deleting *Pten* from granule cells on hippocampal vascular structure, Gli1-CreER^T2+/−^, *Pten*^wt/wt^, TdTom^fl/wt^ (cre control) and Gli1-CreER^T2+/−^, *Pten*^fl/fl^, TdTom^fl/wt^ (DGC-*Pten* KO) mice were generated (Table 1, Experiment #1). DGC-*Pten* KO and control animals were given a subcutaneous injection of tamoxifen (Sigma-Aldrich, T5648; 250 mg/kg dissolved at 20 mg/ml in corn oil) on postnatal day 14 (P14). Blood vessel labeling and tissue collection was conducted when the animals were four, six and 10 weeks old. To label vessels, mice were briefly anesthetized with isoflurane and the left eye was treated with one drop of 0.5% proparacaine hydrochloride ophthalmic solution (AKORN). DyLight 649 labeled Lycopersicon esculentum lectin (Vector Laboratories #DL-1178; 0.1 ml of a 1 mg/ml solution in 10 mm HEPES, 0.1 mm Ca^+2^, 0.15 m NaCl, pH 7.5) was injected into the retro-orbital sinus of the anesthetized eye. Mice recovered for 10–15 min after lectin injections and were then given 0.1 ml of pentobarbital (65 mg/ml) intraperitoneally. When the animals were fully anesthetized, they were transcardially perfused for 1–2 min with 0.1 m PBS with 1 U/ml heparin, followed by a 10 min perfusion with 2.5% paraformaldehyde with 4% sucrose in 0.1 m PBS, pH 7.4. Brains were removed and incubated overnight in the same fixative at 4°C. Brains were cryoprotected in ascending 10%, 20% and 30% sucrose solutions in 0.1 m PBS before freezing. Sagittal cryosections were cut at 60 μm, mounted onto gelatin-coated slides and stored at −80°C.

**Table 1:** Listing of animals used for the five main experiments in the study

Experiment #	Readout	Model	Cellular target	Genotype/AAV	Group	Age (weeks)	Mouse #
1	Vascular structure	DGC-*Pten* KO	≈20% of dentate granule cells	Gli1-CreER^T2+/−^, *Pten^wt^*^/^*^wt^*, TdTom*^fl^*^/^*^wt^*	Control	4	6 (3 F, 3 M)
				Gli1-CreER^T2+/−^, *Pten^wt^*^/^*^wt^*, TdTom*^fl^*^/^*^wt^*	Control	6	7 (2 F, 5 M)
				Gli1-CreER^T2+/−^, *Pten^wt^*^/^*^wt^*, TdTom*^fl^*^/^*^wt^*	Control	10	6 (3 F, 3 M)
				Gli1-CreER^T2+/−^, *Pten^fl^*^/^*^fl^*, TdTom*^fl^*^/^*^wt^*	KO	4	7 (2 F, 5 M)
				Gli1-CreER^T2+/−^, *Pten^fl^*^/^*^fl^*, TdTom*^fl^*^/^*^wt^*	KO	6	8 (4 F, 4 M)
				Gli1-CreER^T2+/−^, *Pten^fl^*^/^*^fl^*, TdTom*^fl^*^/^*^wt^*	KO	10	11 (5 F,6 M)
2	BBB Integrity	DGC-*Pten* KO	≈20% of dentate granule cells	Gli1-CreER^T2−/−^, *Pten^fl^*^/^*^fl^*, tdTom*^wt^*^/^*^wt^*	Control	11	3 (3 F, 0 M)
				Gli1-CreER^T2−/−^, *Pten^fl^*^/^*^fl^*, tdTom*^fl^*^/^*^wt^*	Control	11	2 (1 F, 1 M)
				Gli1-CreER^T2+/−^, *Pten^fl^*^/^*^fl^*, tdTom*^wt^*^/^*^wt^*	KO	11	4 (3 F, 1 M)
3	Vascular structure	FB-*Pten* KO	Forebrain excitatory neurons	CamK2α-Cre^−/−^, *Pten^fl^*^/^*^fl^* (or *Pten^fl^*^/^*^wt^*)	Control	6.5–7	4 (3 F, 1 M)
				CamK2α-Cre^+/−^, *Pten^wt^*^/^*^wt^*	Control	6.5–7	3 (2 F, 1 M)
				CamK2α-Cre^+/−^, *Pten^fl^*^/^*^fl^*	KO	6.5–7	5 (3 F, 2 M)
4	Western blotting	FB-*Pten* KO	Forebrain excitatory neurons	CamK2α-Cre^−/−^, *Pten^fl^*^/^*^fl^*	Control	7–8	2 (1 F, 1 M)
				CamK2α-Cre^+/−^, *Pten^wt^*^/^*^wt^*	Control	7–8	5 (3 F, 2 M)
				CamK2α-Cre^+/−^, *Pten^fl^*^/^*^fl^*	KO	7–8	7 (4 F, 3 M)
5	Vascular structure	f-*Tsc2* KO	Focal loss from cortical excitatory neurons	*Tsc2^wt^*^/^*^wt^* + AAV9-CaMKII- mCherry-Cre	Control	7, 15, 17, 20	4 (2 F, 2 M)
				*Tsc2^fl^*^/^*^fl^* + AAV9-CaMKII- mCherry-Cre	KO	9, 14, 14,15,19	5 (3 F, 2 M)

Experiment #1, DyLight 649 treatment of DGC-*Pten* KOs and controls to examine vascular structure. Experiment #2, AF488-BSA treatment of DGC-*Pten* KOs to assess blood-brain barrier permeability. Experiment #3, DyLight 649 treatment of FB-*Pten* KOs and controls to examine vascular structure. Experiment #4, Western blot analyses of FB-*Pten* KOs and controls. Experiment #5; CD31 immunostaining of focal *Tsc2* KO mice to examine vascular structure.

### Pten immunohistochemistry

Pten immunohistochemistry was performed on study animals to facilitate quantification of the percentage of hippocampal granule cells with *Pten* deletion. Two to four sections per mouse were immunostained with rabbit anti-Pten primary antibodies (1:250, Cell Signaling Technology catalog #9559 RRID:AB_390810), AF647 goat anti-rabbit secondary antibodies (1:750; ThermoFisher Scientific catalog #A32733 RRID: AB_2633282) followed by counterstaining with DAPI. Confocal optical sections of stained dentate gyri were scored to determine the percentage of Pten immunonegative granule cells in accord with established protocols (Arafa et al., 2019; LaSarge et al., 2019, 2021).

### Confocal imaging of vascular structure in DGC-*Pten* KOs

Sagittal brain sections between medial-lateral coordinates 1.56 and 1.8 mm were selected for imaging. Slide-mounted brain sections were thawed, rehydrated, and coverslipped with Prolong Diamond with DAPI (#P36962, Thermo Fisher Scientific). Imaging was performed on a Nikon Ti-E inverted confocal microscope using a 20× objective (NA = 0.75). DyLight 649-lectin was excited with the 647-nm laser line. Sections were imaged through 24 μm of the *z*-depth of the tissue with a 1.0-μm step to create a confocal image stack. Image resolution was 0.63 μm/pixel in the *xy* plane. Images from one section/animal were tiled to capture the entire dentate gyrus.

### Two-dimensional analysis of vascular structure in DGC-*Pten* KOs

To collect vessel area measurements from four-, six-, and 10-week-old control and DGC-*Pten* KO mice, confocal image stacks were converted into maximum projections using Nikon Elements software (AR 5.21.01). The hippocampal fissure and the hippocampal/thalamic border were used as dorsal and ventral boundaries of the dentate gyrus, respectively, while a straight line drawn between the tips of the upper and lower blades of the granule cell body layer set the anterior/posterior boundary. The boundaries of the dentate gyrus were manually encoded using Nikon software, while DyLight 649-lectin labeled vessels were detected automatically using Nikon software’s Object Count function. Identical threshold and parameter settings were used for all slides. Vessel density was calculated by normalizing vessel area to the volume of the dentate gyrus using the following equation: vessel area/(dentate area × 24-μm *z*-depth). Normalizing vessel area to dentate volume is not technically correct, but was felt to be more accurate than normalizing to dentate area, as vessel area measurements were collected from maximum projections of 24-μm-thick dentate image stacks, and thus encompass all vessels in the stack.

### Three-dimensional analysis of vascular structure in DGC-*Pten* KOs

To better elucidate exactly how vessel structure changed in 10-week-old control and DGC-*Pten* KO mice, confocal image stacks through the 24-μm *z*-depth of tissue were imported into Neurolucida 360 software (Microbrightfield) for three-dimensional analysis. Volumetric reconstructions of lectin-stained vessels were used to quantify length, tortuosity, surface area, volume and diameter of hippocampal blood vessels. Quantitative measurements were extracted from the vessel reconstructions using Neurolucida Explorer.

### Permeability assessment in DGC-*Pten* KOs

DGC-*Pten* KO and control mice were generated to determine whether loss of *Pten* alters blood-brain barrier permeability (Table 1, Experiment #2). All *Pten* KO mice and three of five flox control mice were treated with 250 mg/kg of tamoxifen at P14. Alexa Fluor 488 conjugated bovine serum albumin (AF488-BSA, 66,000 Da; Invitrogen #A13100) was given at 11 weeks of age to assess vascular permeability (Marcon et al., 2009; Di Pardo et al., 2017; Ahishali and Kaya, 2021). AF488-BSA was resuspended to 1 mg/ml in sterile normal saline and filtered to remove particulates. Mice were anesthetized with inhaled isoflurane, and 0.1 ml of AF488-BSA injected into the retro-orbital sinus of the left eye as described for tomato lectin. Mice recovered for 10–15 min before euthanizing, perfusing, postfixing and cryoprotecting as described for other animals. Coronal cryosections were cut at 40 μm, mounted onto gelatin-coated slides and stored at −80°C.

To generate positive control animals for blood-brain barrier assessment, pilocarpine status epilepticus was induced in two male 11-week-old Gli1-CreER^T2^−/−, *Pten*^fl/fl^ mice as described previously (Hester et al., 2016; Wulsin et al., 2021). Briefly, mice were injected subcutaneously with 1 mg/kg scopolamine followed 30 min later with 380 mg/kg pilocarpine. Three hours after the onset of status epilepticus, mice were treated with AF488-BSA and perfusion fixed as described for other animals.

Sections were rehydrated in 1× PBS and mounted using Prolong Glass Anti-fade with NucBlue (Thermofisher #P36981). Brain sections from dorsal (bregma = −1.58) and ventral (bregma = −3.1) hippocampi of AF488-BSA infused animals were imaged using a Nikon A1R inverted confocal microscope equipped with a 20× objective (NA = 0.75). Confocal optical sections of the dentate gyri in each section (four to seven dentate gyri/mouse) were collected 5 μm below the tissue surface. AF488-BSA was imaged using the 488 laser line to produce images with a resolution of 1.24 μm/pixel. Images were tiled to capture the entire dentate gyrus. All sections were imaged with identical confocal settings. Images were analyzed using Nikon Elements. A region of interest (ROI) was drawn around the entire dentate gyrus in each confocal optical section, as described for vessel area measurements. Average pixel intensity for regions of interest was calculated and averaged for each animal for statistical analysis.

### Two-dimensional analysis of vascular structure in FB-*Pten* KO mice

To determine whether widespread loss of *Pten* from excitatory neurons in hippocampus and cortex would alter vascular structure, CamK2α-Cre^+/−^, *Pten*^fl/fl^ knock-out mice (FB-*Pten* KOs) were generated. CamK2α-Cre^+/−^, *Pten*^wt/wt^ and CamK2α-Cre^−/−^, *Pten*^fl/fl or fl/wt^ mice served as controls (Table 1, Experiment #3). Additionally, *post hoc* genotype confirmation identified a germline recombination event in two FB-*Pten* KO mice (one male, one female), such that these animals were germline *Pten* heterozygotes combined with CamK2α-Cre mediated recombination of the second *Pten* allele from forebrain excitatory neurons. As this unexpected recombination event might increase the severity of the *Pten* phenotype, potentially providing a more robust test of the role of *Pten* loss in driving vascular changes, these two animals were retained in the study for analysis.

FB-*Pten* KO mice were treated with DyLight 649 lectin and brains prepared for histologic studies when they were 6.5–7 weeks old. FB-*Pten* KO mice undergo significant mortality after 8–10 weeks, so only younger animals can be examined. Vascular labeling and tissue preparation were identical to the procedures described for the two-dimensional analysis of DGC-*Pten* KO mice except that quantification was conducted on the entire hippocampus in each brain slice, as *Pten* loss affects granule cells and pyramidal cells in these animals. The region of cortex immediately above the hippocampus was imaged to assess cortical vasculature. Confocal z-series images collected through 20 μm of tissue at a 1.0-μm step were used for this analysis.

### SDS-PAGE and Western blot analysis in FB-*Pten* KOs

For Western blot studies, a second cohort of FB-*Pten* KO mice was generated (Table 1, Experiment #4). Mice were killed by CO_2_ inhalation between seven and eight weeks of age. Cortex was extracted and immediately frozen on dry ice. Samples were stored at −80°C before tissue lysis.

Tissue lysis was performed with lysis buffer [50 mm Tris (pH 7.4), 40 mm NaCl, 1 mm ethylenediaminetetraacetic acid (EDTA, pH 8), 0.5% Triton X-100, 50 mm NaF, 10 mm Na pyrophosphate, 10 mm Na β-glycerol phosphate, and 1× protease inhibitor; Sigma-Aldrich, catalog #11873580001]. Protein concentration was determined using Bio-Rad Protein Assay Dye (catalog #5000006). Samples were mixed with SDS sample buffer and equal amounts of proteins were run in duplicate on SDS-PAGE gels and transferred to PVDF Transfer Membranes (Millipore Sigma). Membranes were blocked using 5% milk for 1 h. The following antibodies were used for Western blotting: Pten (1:1000; catalog #9559, RRID: AB_390810), anti-β-Actin (1:40,000, Sigma #A1978, clone AC-15, RRID:AB_476692), and vascular endothelial growth factor A (VegfA; 1:1000; Abcam, catalog #ab46154, RRID: AB_2212642). Antibodies were diluted to the desired concentration in 1% Tween in 0.1M PBS and incubated at 4°C overnight. Membranes were then washed and incubated with secondary antibody; either Rabbit IgG HRP Linked Whole Antibody (1:2000; Millipore Sigma; catalog #GENA934) or Mouse IgG HRP Linked Whole Antibody (1:2000; Millipore Sigma; catalog #NXA931V). Signals were detected with enhanced chemiluminescence using Pierce ECL Blotting Substrate (Thermo Scientific, catalog #32106). If a second detection was needed, blots were stripped using Restore Western Blot Stripping buffer (Thermo Scientific, catalog #21059), blocked again in 5% milk, and incubated overnight with the desired antibody. Specific signals on Western blottings were quantified by densitometry using NIH ImageJ software. Signal intensities were normalized to β-Actin on the same blot. Duplicate samples were averaged for statistical analysis.

### Two-dimensional analysis of vascular structure in cortical f-*Tsc2* KO mice

To determine whether focal loss of *Tsc2* from excitatory neurons in cortex would alter vascular structure, *Tsc2*^fl/fl^ and *Tsc2*^wt/wt^ mice were generated (Table 1, Experiment #5). All mice received two bilateral stereotaxic injections starting with the left hemisphere (four injections total at coordinates A/P, M/L, D/V: 0.5, ±0.5, −0.35 and −1.0, ±0.65, −0.35). Injections contained AAV9-CaMK2α_short>mCherry:T2A:Cre:WPRE vector with 0.05% Trypan Blue in sterile PBS (50 nl per site of 3.55x 10^9^ gc/μl concentration; VectorBuilder, VB200130-1243kmp) and were conducted on P2 using hypothermia anesthesia (Kim et al., 2014). Pups were returned to the mother after the injection and weaned at P28. As part of another experiment, two *Tsc2*^wt/wt^ mice and four *Tsc2*^fl/fl^ underwent EEG implantation surgery between 12 and 13 weeks of age in accord with established protocols (LaSarge et al., 2021). All mice were perfused between seven and 20 weeks of age (control: 104 ± 18.9 d old; f-*Tsc2* KOs: 100 ± 11.0 d; mean ± SEM; Table 1, Experiment #5). Tissue was postfixed, cryoprotected, frozen, cut, and stored as described for other animals.

### Tsc2 immunohistochemistry

Tsc2 immunohistochemistry was performed on tissue from study animals to verify loss of Tsc2 in virally-infected (mCherry-expressing) cells. Sections were immunostained with rabbit anti-Tuberin/Tsc2 primary antibodies (1:750, Cell Signaling Technology catalog #4308 RRID:AB_10547134), AF647 goat anti-rabbit secondary antibodies (1:750; Thermo Fisher Scientific catalog #A32733 RRID: AB_2633282) and counterstained with NeuroTrace 500/525 (1:300; Invitrogen catalog #N21480). Slides were mounted with Prolong Glass Anti-fade with NucBlue (Thermofisher #P36981). Images were acquired using a Nikon AXR inverted confocal microscope equipped with a 10× (NA = 0.45) and 60× water immersion (NA = 1.20) objective.

### Analysis of vascular structure in f-*Tsc2* KO mice

To quantify blood vessel density, CD31 immunohistochemistry was conducted. Bregma-matched tissue sections from control and f-*Tsc2* KO mice (A/P 1.4-2.4) were immunostained with rat-anti mouse CD31 primary antibody (1:500, BD Biosciences catalog #553370) and AF647 goat anti-rat secondary antibodies (1:750, Thermo Fisher Scientific catalog #A21247 RRID: AB_2633282). The slides were coverslipped with ProLong Glass Antifade Mountant with NucBlue (#P36981, Thermo Fisher Scientific). Slides were then screened to identify mCherry expressing regions in cortex. Injections in the left hemisphere consistently produced focal mCherry labeling in cortex, while mCherry labeling was rare in the right hemisphere, likely a consequence of the syringe clogging after the first one to two injections. Imaging was performed on a Nikon Ti-E inverted confocal microscope using a water immersion 20× objective (NA = 0.95). Serial channel imaging was used to capture mCherry-labeled cells (581 nm) and CD31 expression (647 nm) in a 635 × 635 (*xy*) × 12 (*z*-depth, 0.5-μm step) field centered on the region containing mCherry-labeled *Tsc2* KO or control cells in the left hemisphere. An anatomically correspondent region of interest (ROI) in the right hemisphere lacking mCherry expression was also imaged using identical settings. Confocal image stacks were converted into maximum projections using Nikon Elements software (AR 5.21.01). Vessel density in the right hemisphere was determined using Nikon software’s Object Count function. For the left hemisphere, images were imported into Neurolucida 360 software to define a ROI surrounding mCherry-expressing *Tsc2* KO cells, or mCherry-expressing wild-type cells in control mice. The user-guided tracing function was employed to trace the blood vessels within each ROI. Vessel area was normalized to ROI volume for statistical analyses, as described for the DGC-*Pten* KO model.

### Statistical analysis

All data collection was conducted by investigators unaware of animal genotype or treatment group. Statistical significance was determined using Sigma Plot (version 14), R or GraphPad Prism (version 9.3.1) with α = 0.05. Sex effects were assessed by including sex as a factor in two-way or three-way ANOVAs. No effects of sex were found except as noted. Note as well that the study was not powered to detect sex differences. In the absence of differences, data from male and female animals was pooled. Data were tested for normality and equal variance, and parametric or nonparametric equivalents used as appropriate. Blood-brain barrier leakage was assessed using a linear mixed effect model with a random animal effect. Values presented are mean ± SD unless otherwise noted.

## Results

To determine whether loss of the mTOR negative regulator *Pten* from a subset of dentate granule cells induces vascular changes in the dentate gyrus, a conditional, inducible mouse model approach was used to delete *Pten* from hippocampal dentate granule cell progenitors (DGC-*Pten* KOs; Table 1, Experiment #1). To induce *Pten* deletion, mice were treated with tamoxifen on P14, when many granule cell progenitors are still active. Tamoxifen-activated cre recombinase leads to the deletion of *Pten* and expression of tdTomato among hippocampal granule cell progenitors and all subsequent daughter cells (Pun et al., 2012; LaSarge et al., 2015, 2016). Among DGC-*Pten* KO animals, 22% (8 of 36) died before the experimental endpoint compared with 0 of 21 control animals. Mice were harvested at four, six, or 10 weeks of age (two, four, and eight weeks after tamoxifen treatment). On the day of harvest, mice were injected retro-orbitally with a fluorescent lectin (DyLight 649) to label the vasculature.

### DGC-*Pten* KO cell load

**Figure 1.**
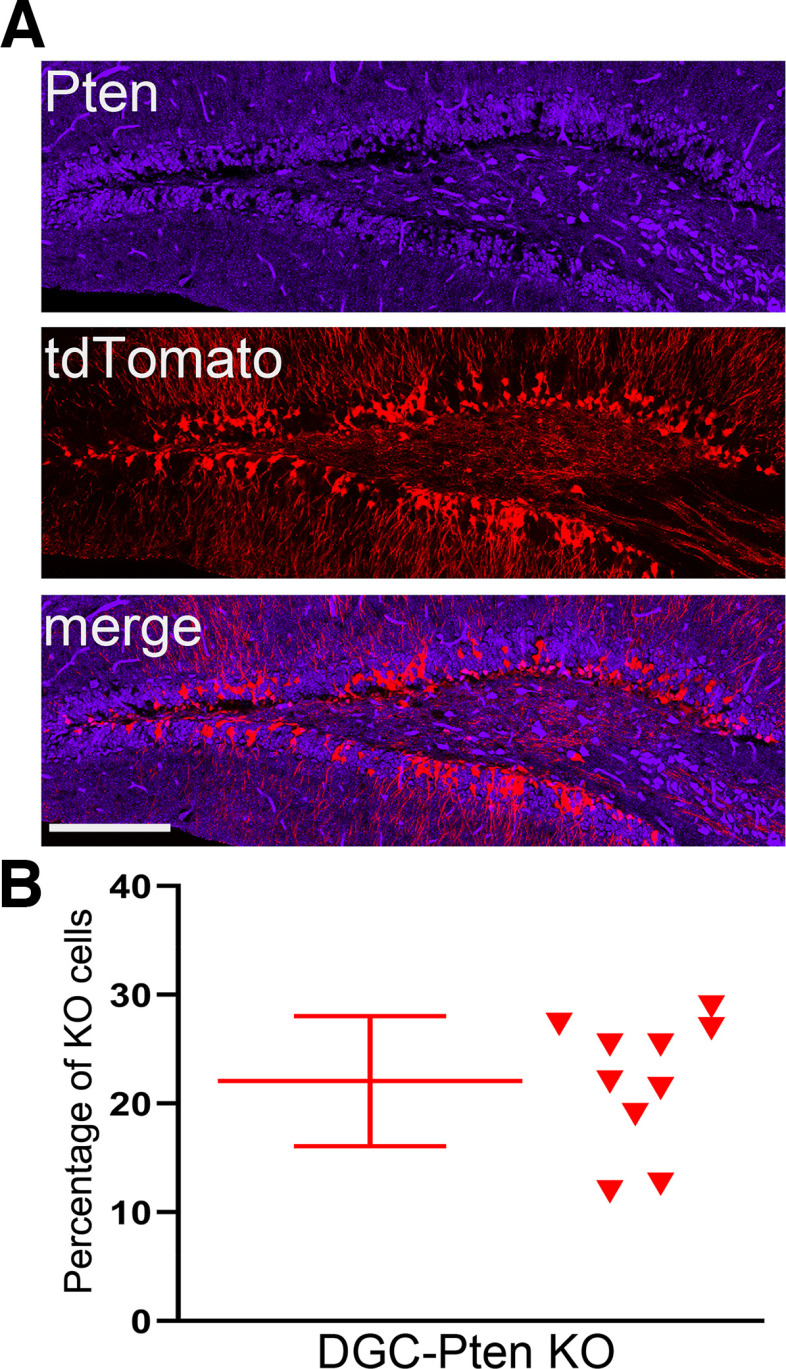
***A***, Pten immunostaining (blue) and tdTomato fluorescence (red) in a 10-week-old DGC-*Pten* knock-out (KO) mouse. Scale bar = 100 μm. ***B***, Scatterplot showing the percentage of DGC-*Pten* KO cells for a subset of animals used in the present study.

In prior studies, we have observed that animals with DGC-*Pten* KO cell loads >10% exhibit spontaneous cortical seizures as early as eight weeks (Pun et al., 2012). As seizures can drive changes in microvascular density (van Lanen et al., 2021), the current study was designed to produce animals with KO rates in excess of 10%. Quantification of DGC-*Pten* KO cell number in 10-week-old KO animals revealed that, on average, 22.1 ± 6.0% of granule cells lacked Pten protein (*n* = 10; range 11.9–28.9%; Fig. 1). Although seizures were not directly assessed here, animals in the current study were well above the threshold for spontaneous cortical seizures established in prior work (Pun et al., 2012; LaSarge et al., 2021).

### DGC-*Pten* KO mice exhibit increased hippocampal vasculature but preserved vascular density

**Figure 2.**
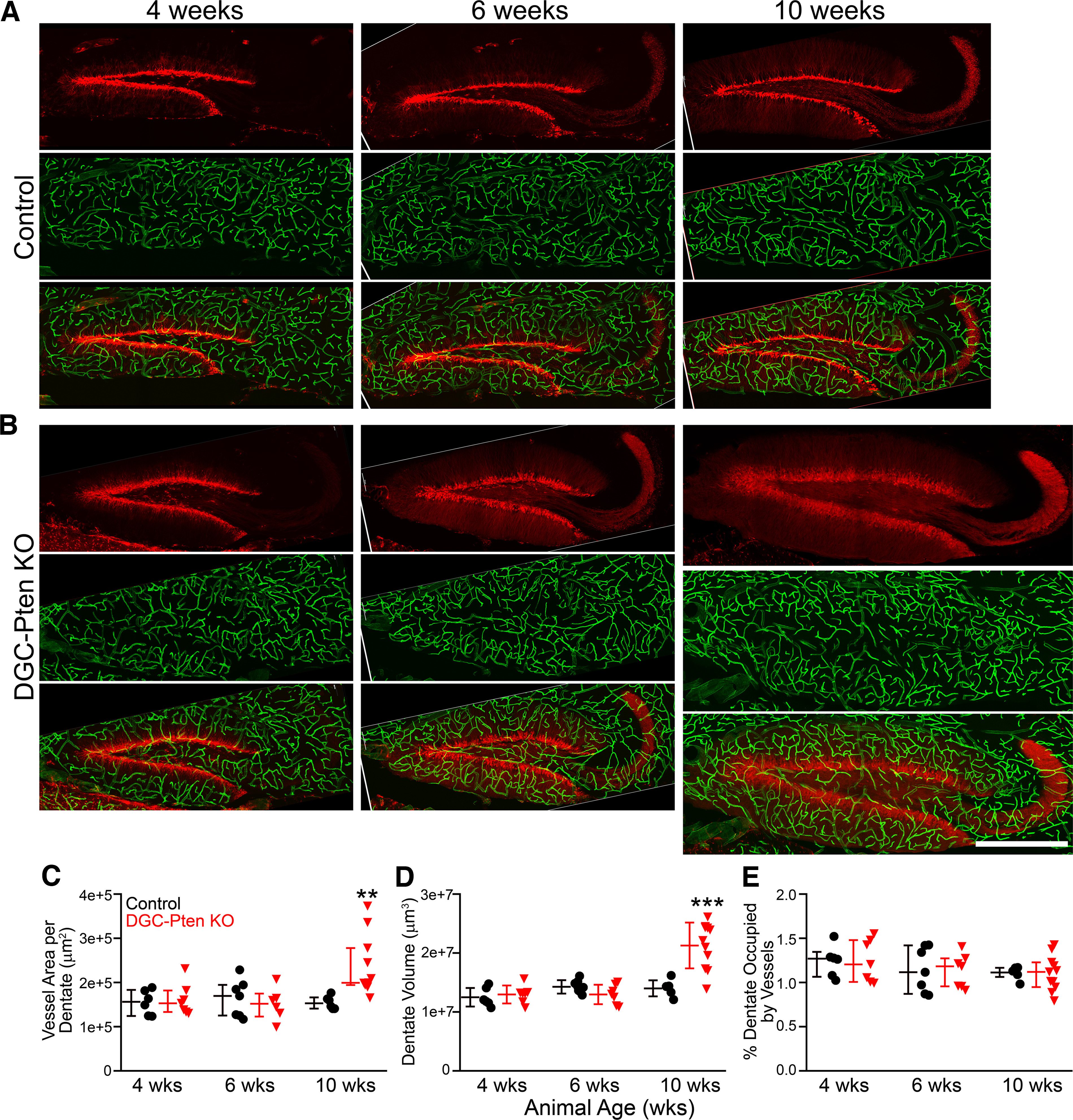
***A***, ***B***, Confocal images of hippocampal dentate gyri showing granule cells expressing tdTomato (red) and blood vessels labeled with DyLight 649-lectin (green). Dentate gyri from four-, six-, and 10-week (wks) control (***A***) and DGC-*Pten* knock-out (KO) (***B***) animals are shown. All images are at the same scale, demonstrating the striking hypertrophy of the dentate in the 10-week KO. Scale bar = 500 μm. ***C***, Scatterplots of individual animal scores and group medians ± interquartile range for vessel area in control (black) and DGC-*Pten* KO (red) animals. Area was calculated from maximum projections of images from 24-μm-thick hippocampal sections. ***D***, Scatter plot and group means ± SD for the volume of the dentate gyrus in 24-μm-thick hippocampal sections. ***E***, Vessel area (***C***) was normalized to dentate volume (***D***) to estimate the percentage of the dentate occupied by vessels in 24-μm-thick hippocampal slices. Values are group medians ± interquartile range. **p < 0.01, ***p < 0.001.

Hippocampi from four, six and 10-week-old control and DGC-*Pten* KO mice were examined to determine total vessel area in the dentate gyri of hippocampal sections (Fig. 2*A*,*B*). Among control animals, dentate vessel area was stable across all three time points (two-way ANOVA on log transformed data; 4 vs 6, *p* = 0.944; 4 vs 10, *p* = 0.999; 6 vs 10, *p* = 0.961; Fig. 2*C*). Among KOs, by contrast, vessel area was significantly greater at 10 weeks relative to four (*p* = 0.004) and six (*p* < 0.001) weeks. Vascular area in the 10-week KO group was 51.9% greater than the 10-week control group (*p* < 0.001). These data demonstrate that vascular area expands significantly in DGC-*Pten* KO animals between six and 10 weeks.

To determine whether the observed increase in vessel area also produced an increase in vessel density, we quantified dentate volume for each brain slice examined. *Pten* loss can increase brain size (Kwon et al., 2003) driven at least in part by somatic and dendritic hypertrophy of KO cells (LaSarge et al., 2015, 2019; Santos et al., 2017). Visual examination of dentate gyri from 10-week KO animals revealed that the dentate gyrus was indeed larger (Fig. 2*A*,*B*). Quantification of dentate volume in each brain slice revealed changes that paralleled vessel area. Dentate volume was stable across all three time points in control mice (two-way ANOVA on log transformed data; 4 vs 6, *p* = 0.198; 4 vs 10, *p* = 0.325; 6 vs 10, *p* = 0.966; Fig. 2*D*). Among KOs, by contrast, dentate volume was significantly greater at 10 weeks relative to four (*p* < 0.001) and six (*p* < 0.001) weeks. Dentate volume in the 10-week KO group was 51.8% greater than the 10-week control group (*p* < 0.001). To approximate vessel density, we normalized vessel area to dentate volume for each brain slice. Vessels occupied ∼1% of the dentate volume in this analysis. No differences in vessel density were evident among different ages (two-way ANOVA on log transformed data, *p* = 0.170; Fig. 2*E*) or genotypes (*p* = 0.941). Taken together, these findings indicate the DGC-*Pten* KO animals exhibit ∼50% increases in blood vessel area and dentate volume, but overall vessel density is preserved. Therefore, although DGC-*Pten* KO dentate gyri are larger in 10-week animals, the data suggest that the average distance between a neuron and the nearest vessel is unchanged.

### Volumetric analyses of vascularity in DGC-*Pten* KO mice

Given the robust changes in dentate structure in 10-week DGC-*Pten* KOs, we sought to analyze vessel structure in these animals in greater detail. Three dimensional reconstructions of vessels in each dentate were generated, encoding vessel length, volume, diameter, tortuosity and volume of the dentate slice sampled. This approach allows us to calculate a true vessel density relative to the area measurements and estimates shown in Figure 2.

**Figure 3.**
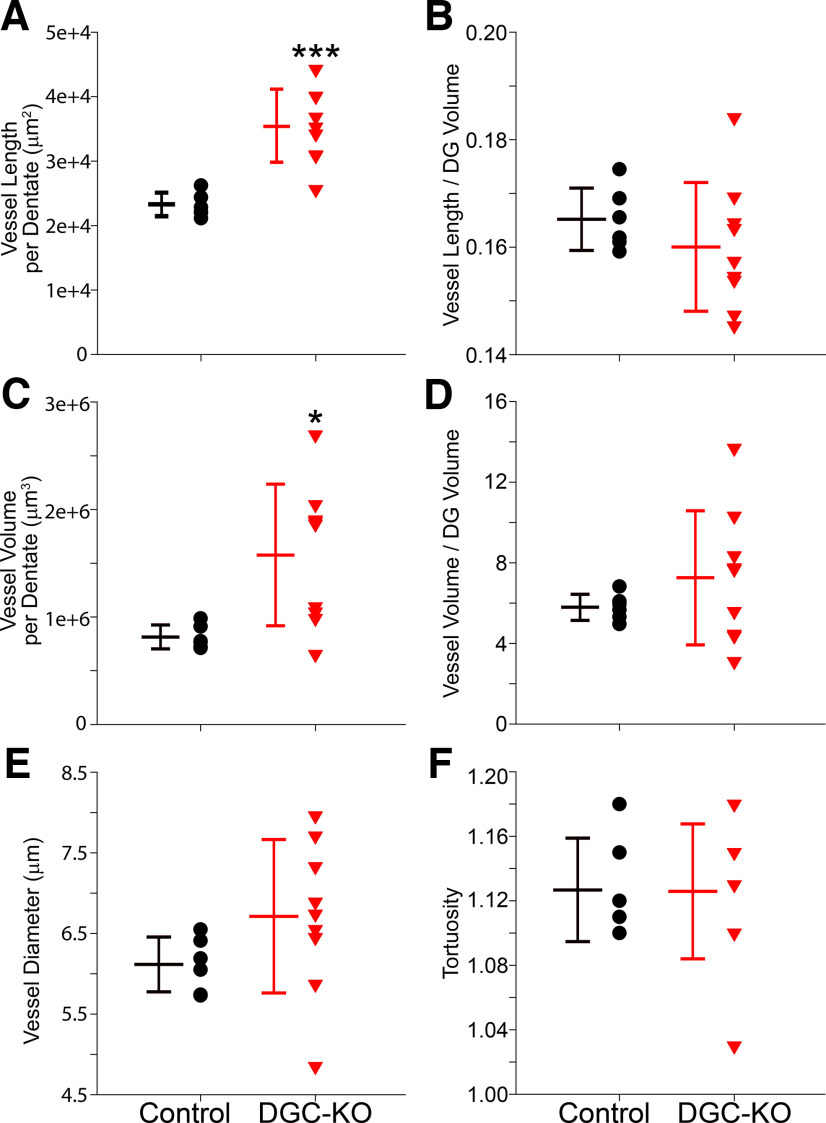
Graphs show scatterplots of individual animal scores and group means ± SD for 10-week control and DGC *Pten*-KO animals. All data were collected from a 24-μm-thick section of the hippocampal dentate gyrus (DG). ***A***, Mean vessel length per dentate section. ***B***, Mean vessel length per dentate brain section divided by the volume of the section X 100. ***C***, Mean vessel volume per dentate section. ***D***, Mean vessel volume per dentate brain section divided by the volume of the section X 100. ***E***, Mean vessel diameter. ***F***, Mean vessel tortuosity. *p < 0.05, ***p < 0.001.

Vessel length per dentate was significantly increased in 10-week DGC-*Pten* KOs relative to 10-week controls (control, *n* = 6; KO, *n* = 9; Welch’s *t* test, *p* < 0.0001; Fig. 3*A*). When normalized to dentate volume, however, no differences in overall vessel density were evident (*t* test, *p* < 0.346; Fig. 3*B*). Similar findings were evident when vessel volume was examined. Total vessel volume per dentate was significantly increased in KOs (Welch’s *t* test, *p* = 0.008; Fig. 3*C*), but vessel volume normalized to dentate volume was unchanged (Welch’s *t* test, *p* = 0.234; Fig. 3*D*). Vessel diameter was statistically similar between groups (*t* test, *p* = 0.170; Fig. 3*E*). Finally, we compared vessel tortuosity, a measure of how “twisted” a vessel’s path is. Tortuosity was not found to differ among groups (*t* test, *p* = 0.957; Fig. 3*F*). These findings confirm the increase in vessel extent per dentate observed in the prior analysis, and the preservation of overall vessel density. They further reveal that increasing vessel extent per dentate is driven by vessel lengthening, without overt changes in vessel diameter or path, at least at the time points examined.

### Blood-brain barrier leakage

Seizures can induce blood-brain barrier leakage (Gorter et al., 2019). Angiogenesis can also be associated with impaired blood-brain barrier integrity (Rust, 2020). To determine whether the blood-brain barrier might be impaired in DGC-*Pten* KO mice, a new group of 11-week-old animals was treated with AF488-conjugated bovine serum albumin (BSA) before perfusion (Table 1, Experiment #2). The conjugated albumin is too large to pass through the wall of blood vessels, so its presence in the extra-vascular space is indicative of alterations in blood-brain barrier permeability. AF488-BSA is cleared from within vessels during perfusion, so only reagent that leaked out of the vessels is retained. Analyses focused on the dentate gyrus, where *Pten* KO cells are localized.

**Figure 4.**
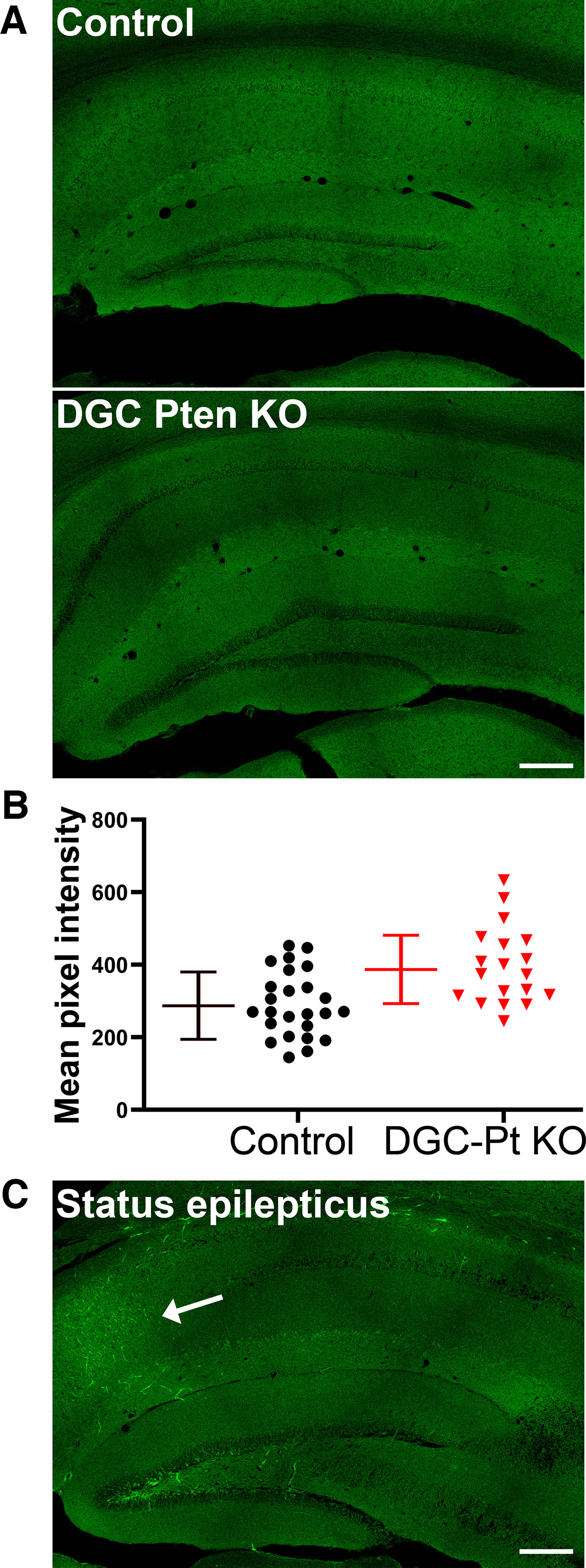
***A***, AF488-BSA fluorescence in the hippocampi of a control mouse and a DGC-*Pten* (Pt) KO mouse. No evidence of extravascular AF488-BSA was seen in either group. Scale bar = 200 μm. ***B***, Symbols denote the mean pixel intensity of the AF488-BSA signal for each control and DGC-*Pten* KO dentate gyrus brain section. Bars show animal means and SD (*N* = 5 controls and 4 DGC-*Pten* KOs, 4–7 sections/mouse; *p* = 0.1195). ***C***, AF488-BSA fluorescence in the hippocampus of a mouse that went through pilocarpine-induced status epilepticus. This positive control animal shows a clear increase in AF488-BSA labeling in the CA1 pyramidal cell layer (arrow). Scale bar = 200 μm.

Gross examination of brain slices from DGC-*Pten* KO mice revealed no evidence of blood-brain barrier leakage relative to controls (Fig. 4*A*). In addition to gross analyses, mean AF488-BSA signal intensity was collected from four to seven hippocampi per DGC-*Pten* KO or control mouse. Although a trend toward slightly increased signal intensity was evident among DGC-*Pten* KO mice, the effect did not reach significance (Control, pixel intensity = 286 ± 93; *Pten* KO, 386 ± 95, Linear mixed effect model with a random animal effect, *p* = 0.1195; Fig. 4*B*).

To confirm the efficacy of AF488-conjugated BSA in detecting blood-brain barrier leakage, two DGC-*Pten*^fl/fl^ control mice were infused with the conjugate immediately after 3 h of pilocarpine-induced status epilepticus. Modest leakage was evident in the CA1 region and hilus of one of the mice (Fig. 4*C*), while the second exhibited patchy regions of intense AF488 BSA accumulation in cortex and thalamus. No leakage was evident in the dentate cell body layer in these animals, although it is noted that granule cells are relatively resistant to damage in this model. These findings demonstrate that AF488-conjugated BSA infusion can reveal blood-brain barrier leakage, and support the conclusion that increased vascular permeability is not a prominent or persistent feature of the DGC-*Pten* KO mouse model of epilepsy.

### Vascular changes in forebrain-specific *Pten* KO mice

The absence of an effect on vascular density in DGC-*Pten* KO mice could reflect the relatively small population of affected neurons (22% of granule cells). We queried, therefore, whether *Pten* deletion from forebrain would impact vascular structure by examining 6.5- to 7-week-old CamK2α-Cre, *Pten*^fl/fl^ mice (FB-*Pten* KOs; Table 1, Experiment #3). CamK2α-Cre drives widespread *Pten* deletion from excitatory neurons in cortex and hippocampus, a much broader cellular distribution than the DGC-*Pten* KOs.

FB-*Pten* KO mice had enlarged overall brain weights relative to controls (control, 0.377 ± 0.030 g; FB-*Pten* KO, 0.447 ± 0.026 g; two-way ANOVA with genotype and sex as factors, *p* = 0.002). There was no significant effect of sex (*p* = 0.505) or interaction between factors (*p* = 0.094). When brain weight was expressed as a percentage of body weight, significant effects of genotype (*p* = 0.029) and sex (*p* = 0.016) were found (Control male, 1.7 ± 0.0%; FB-*Pten* KO male, 2.2 ± 0.1; Control female, 2.2 ± 0.2; FB-*Pten* KO female, 2.3 ± 0.2). There was no significant interaction between sex and genotype (*p* = 0.105). These findings demonstrate that *Pten* deletion from forebrain produces an overall gross disruption of brain size.

**Figure 5.**
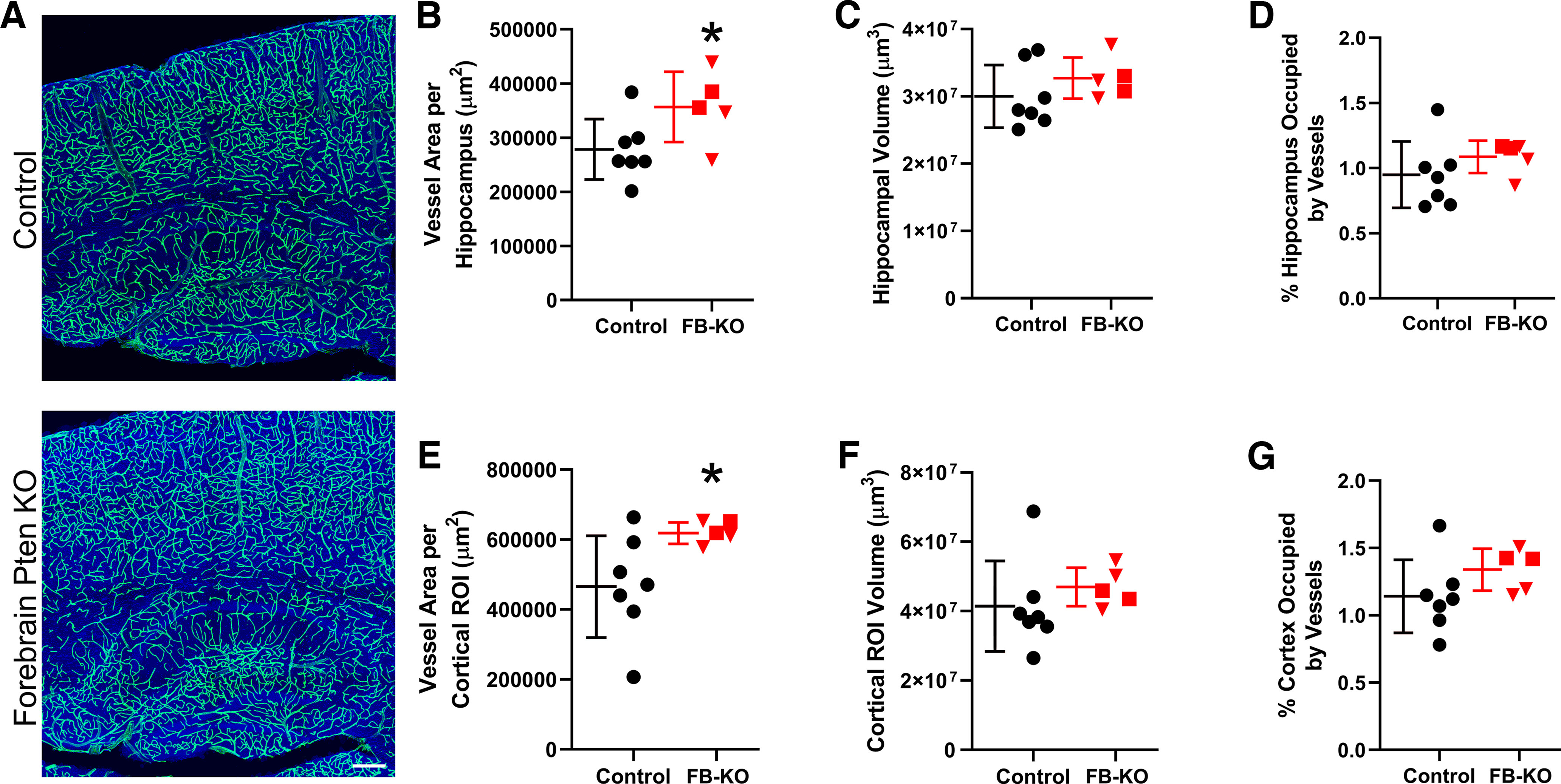
***A***, Confocal images of hippocampus and cortex showing blood vessels labeled with DyLight 649-lectin (green). The tissue is counterstained with nuclear blue. Scale bar = 200 μm. ***B***, ***E***, Graphs show scatterplots of individual animal scores and group means ± SD for vessel area in control (black) and Forebrain (FB)-*Pten* KO (red) animals for hippocampus (***B***) and cortex (***E***). Area was calculated from maximum projections of images from 20-μm-thick sections. ***C***, ***F***, Scatter plot and group means for the volume of the hippocampus (***C***) and cortex (***F***) in 20-μm-thick sections. ***D***, ***G***, Vessel area (***B***, ***E***) was normalized to dentate volume (***C***, ***F***) to estimate the percentage of the hippocampus (***D***) or cortex (***G***) occupied by vessels in 20-μm-thick slices. Red squares denote the two CamK2α-Cre^+/−^, *Pten*^fl/-^ mice with germline loss of one *Pten* allele. *p < 0.05.

Quantification of vascular area was conducted for hippocampus and cortex, both regions with widespread *Pten* loss. Within the hippocampus, vessel area showed a significant 28.5% increase in FB-*Pten* KOs relative to controls (*t* test, *p* = 0.049; Fig. 5). Similarly, vessel area in cortex was increased by 33.1% in FB-*Pten* KOs relative to controls (*t* test, *p* = 0.045). As with DGC-*Pten* KOs, vessel area in FB-*Pten* KOs was normalized to the volume of tissue examined to determine whether there was a net increase in vessel density. Neither hippocampal (*t* test, *p* = 0.283; Fig. 5) nor cortical (Mann–Whitney rank-sum test, *p* = 0.106) volume was significantly increased, although results in cortex should be interpreted cautiously, as it was not possible to use the natural boundaries of the structure. When vessel area was normalized to the volume of each sample region (Fig. 5), no increase in vessel density was evident for either hippocampus (*t* test, *p* = 0.149) or cortex (*t* test, *p* = 0.176). In addition, values from two FB-*Pten* KOs with the unexpected germline loss of one *Pten* allele (Fig. 5, red squares) overlapped with values from animals with *Pten* loss restricted to forebrain (Fig. 5, red triangles), suggesting that global *Pten* heterozygosity does not amplify the phenotype. The findings indicate that even widespread loss of *Pten* from neurons is not sufficient to drive robust hypervascularization.

### Vascular endothelial growth factor (VegfA)

**Figure 6.**
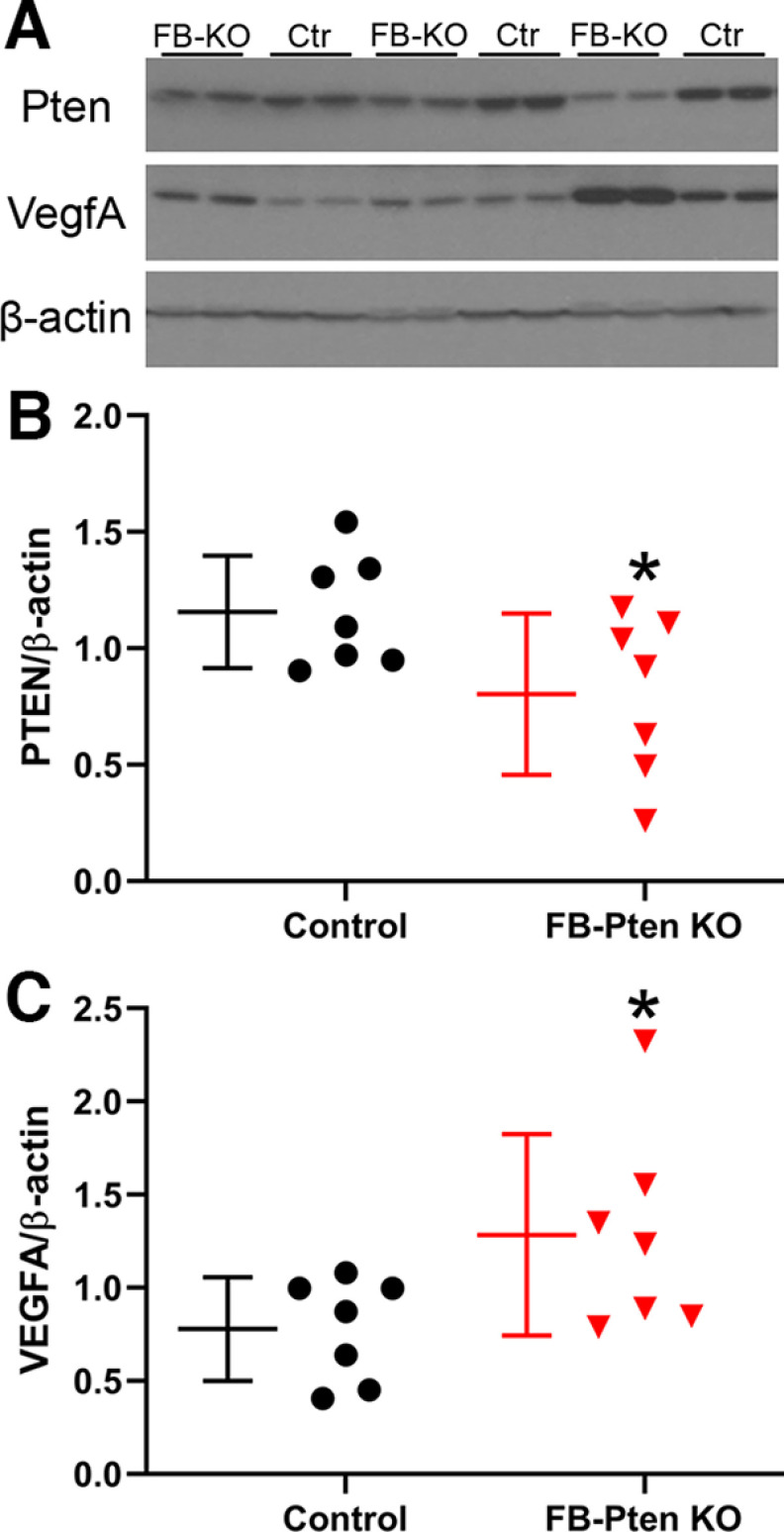
VegfA is increased in mice with neuronal *Pten* loss from forebrain (FB-KO). ***A***, Representative Western blottings of samples from FB-KO and littermate control (Ctr) cortex. ***B***, Pten protein is reduced in the cortex of FB-KO mice. ***C***, VegfA protein is increased in FB-KO mice. Each point represents averaged data from single mouse. *p < 0.05.

Vascular remodeling and angiogenesis can be driven by increased expression of angiogenic factors known to be downstream targets for mTOR, including VegfA. We queried, therefore, whether neuronal *Pten* loss drives increased VegfA expression in FB-*Pten* KO mice. Western blot analyses were conducted using a second cohort of control and FB-*Pten* KO mice (Table 1, Experiment #4). Western blot analyses confirmed a 31% decrease in Pten in the cortex of FB-*Pten* KO mice compared with littermate controls (*t* test, *p* = 0.047; Fig. 6). VegfA protein levels, on the other hand, were increased by 65% in FB-*Pten* KOs relative to controls (*t* test, *p* = 0.048; Fig. 6). Findings support the conclusion that neuronal *Pten* deletion can drive increased VegfA protein levels.

### Focal *Tsc2* loss from cortical excitatory neurons increases vascular density

**Figure 7.**
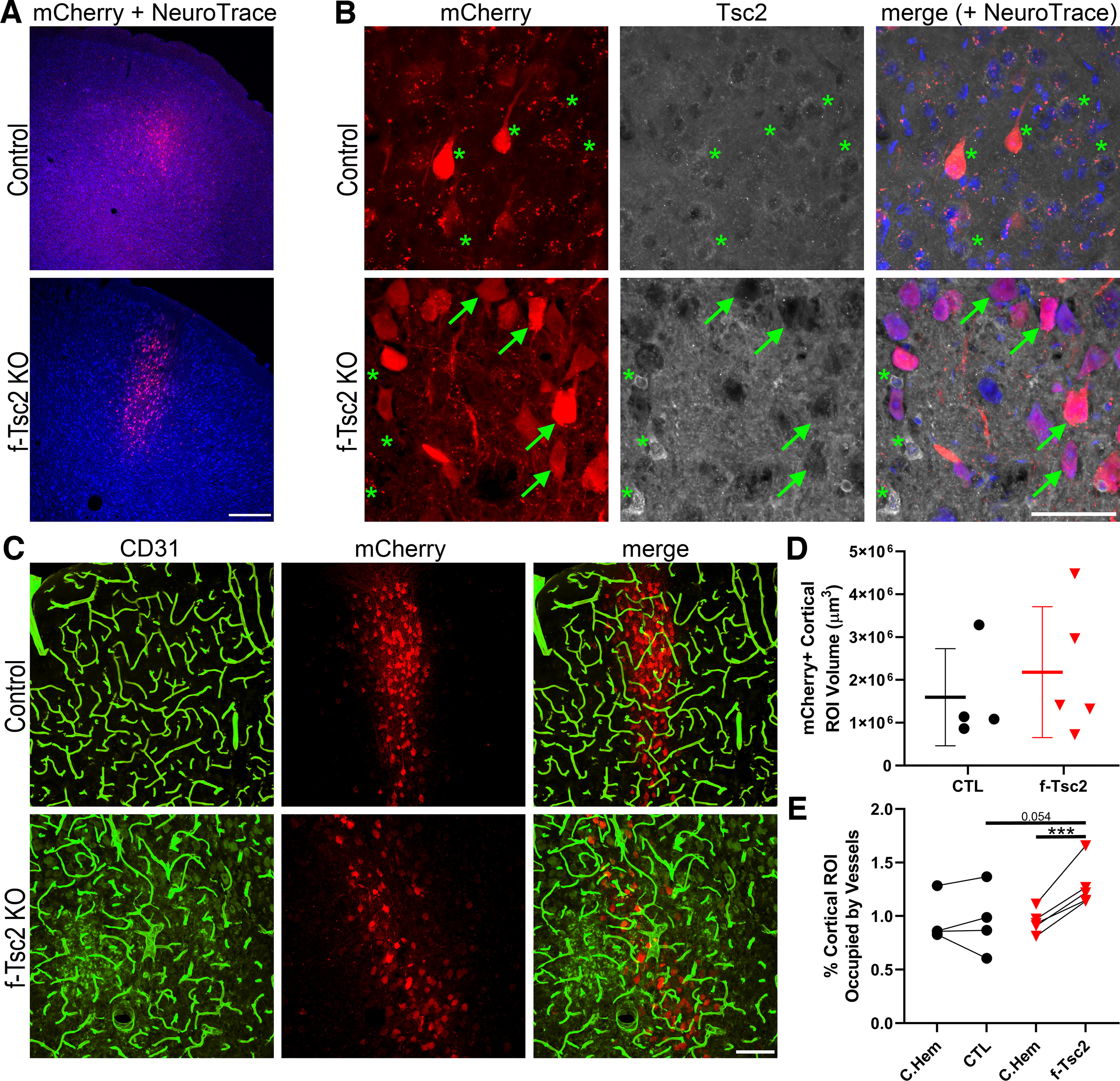
***A***, Confocal optical sections of cortex showing focal mCherry expression (red) with a NeuroTrace counterstain (blue) in control (*Tsc2*^wt/wt^) and f-*Tsc2* KO (*Tsc2*^fl/fl^) mice injected with AAV9-Cre-mCherry. Scale bar = 300 μm. ***B***, Confocal maximum projections (4-μm depth) of mCherry-expressing cells in control and f-*Tsc2* KO cortical tissue immunostained for Tsc2. Cells in control animals, and mCherry negative cells in KOs (*) show cytoplasmic Tsc2 labeling, with exclusion of the protein from the nucleus. Staining is absent from mCherry-expressing cells in KOs (arrows). Scale bar = 50 μm. ***C***, Blood vessels immunostained with CD31 (green) are shown around *Tsc2* KO cells labeled with mCherry (red) in a f-*Tsc2* KO mouse, while mCherry-labeled wild-type neurons are shown in the control mouse. Scale bar = 100 μm. ***D***, Graph shows the area occupied by mCherry-expressing (*Tsc2* KO, red) neurons in the cortex of f-*Tsc2* KO mice relative to the area occupied by mCherry-expressing (wild-type, black) neurons in control (CTL) mice. ***E***, Graph shows the percentage of cortical regions of interest (ROIs) occupied by blood vessels for control and f-*Tsc2* KO mice [(vessel area/cortical ROI volume)*100]. Each paired set of points represents one animal, with the first point giving vessel density in the mCherry-negative right hemisphere (C. Hem), while the second point is the measure for the mCherry-expressing left hemisphere. ***p < 0.001.

To query whether focal loss of *Tsc2* might produce a different effect than *Pten* loss, *Tsc2^fl/fl^* mice received bilateral cortical injections of AAV9-CaMKII-mCherry-T2A-Cre on postnatal day 2, leading to mCherry expression and cre-mediated focal loss of *Tsc2* from cortical excitatory neurons in *Tsc2*^fl/fl^ mice, and mCherry expression-only in *Tsc2*^wt/wt^ control mice. Despite bilateral injections, mCherry expression was restricted to the left hemisphere in most animals, likely because of clogging of the syringe used to inject the AAV. In the left hemisphere, mCherry expression tended to be localized to upper layers of cortex, although variability among animals was noted (Fig. 7*A*,*B*). The volume occupied by mCherry-expressing neurons in the 12-μm-thick confocal image stacks examined averaged 1,594,609 μm^3^ in control animals, and 2,178,774 μm^3^ in f-*Tsc2* KO animals (*t* test, *p* = 0.546, *t* test; Fig. 7*C*,*D*), confirming that viral injections were similar across groups. To determine whether *Tsc2* KO lesions exhibited increased vascularity, within animal analyses comparing mCherry-expressing cortical regions in the left hemisphere to anatomically comparable mCherry-negative regions in the right hemisphere were conducted for both genotypes [*f-Tsc2 KO* and control (*Tsc2*^wt/wt^)], revealing a significant interaction between genotype and hemisphere [two-way repeated measures ANOVA controlling for animal, genotype and hemisphere (as the repeated measure); *p* = 0.008; Fig. 7*E*]. *Post hoc* tests showed significantly increased vascular density in the mCherry-expressing hemisphere relative to the mCherry-negative hemisphere within f-*Tsc2* KOs (Bonferroni *t* test, *p* < 0.001) but not controls (*p* = 0.993). Comparisons across animals showed a similar pattern. Specifically, brain hemispheres lacking mCherry expression did not differ significantly between control and f-*Tsc2* KO mice (*p* = 0.948), while a nonsignificant trend was evident when mCherry-expressing hemispheres from f-*Tsc2* KOs were compared with mCherry-expressing hemispheres from control mice (*p* = 0.054).

## Discussion

Mutations leading to excess mTOR pathway signaling have been associated with vascular abnormalities. Here, we examined two mouse models with either focal hippocampal or widespread forebrain loss of the mTOR negative regulator *Pten*, and a third model with focal cortical loss of the mTOR negative regulator *Tsc2*. In both *Pten* models, vessel area was increased. *Pten* deletion, however, also led to gross increases in brain volume, and when vessel areas were normalized to these larger volumes, overall vessel density was found to be preserved. In addition, although the angiogenic factor VegfA was increased in FB-*Pten* KOs, the absence of hypervascularization suggests this increase may simply reflect enhanced vessel growth to accommodate larger brain volumes. By contrast, focal loss of *Tsc2* from excitatory cortical neurons significantly increased vessel density within the KO region. Together, these findings suggest that neuronal *Tsc2* loss may exert a greater effect on tissue vascularity than *Pten* loss, however, further studies are needed to exclude potential model-specific differences. Regardless, studies demonstrate that hypervascularization is not a ubiquitous consequence of enhanced mTOR pathway signaling in brain.

### Regulation of angiogenesis by different mTOR pathway genes

A plausible explanation for the different effects of *Pten* versus *Tsc2* deletion is that distinct mTOR pathway genes differentially regulate angiogenesis. Tsc1, Tsc2 and Pten occupy different positions in the mTOR signaling cascade (Pten is upstream of Tsc1/2 and Rheb) and distinct proteins can have mTOR independent effects. Indeed, Tsc2 may be able to regulate VegfA through mTOR-independent mechanisms (Brugarolas et al., 2003). It would not be particularly surprising, therefore, for mutations in different mTOR pathway genes to produce distinct effects on angiogenesis. *Pten*, however, has been shown to directly regulate angiogenesis in glioma models (Wen et al., 2001) and patients with *Pten* mutations often exhibit vascular abnormalities (Dhamija and Hoxworth, 2020). To further query whether *Pten* deletion could drive angiogenesis, we examined VegfA protein levels in FB-*Pten* KO mice and found that *Pten* deletion did result in increased amounts of VegfA protein. The finding confirms that *Pten* deletion does increase this major angiogenic factor, but perhaps only to drive sufficient angiogenesis to accommodate the larger brain volumes. By contrast, the present findings with *Tsc2* now add to an expanding body of research linking the Tsc1/Tsc2 complex to disrupted brain vascularity. Specifically, *Tsc1* deletion from cortical excitatory neurons and astrocytes using *Tsc1^fl/fl^*, Emx1-Cre mice and germline loss of *Tsc2* from Eker rats produces mTOR hyperactivation and vascular abnormalities (Zhang et al., 2019; Kútna et al., 2020). In utero electroporation of a constitutively active Rheb, the direct target of the Tsc1/Tsc2 complex, also causes hypervascularity (Zhang et al., 2019).

In addition to the genes themselves, the cell types targeted for gene deletion could also be a critical variable. Zhang and colleagues were able to target a variety of cortical neurons and some astrocytes with their approaches (Zhang et al., 2019), whereas the Gli1-CreER^T2^ driver used in DGC-*Pten* KO model targets hippocampal granule cells through the rostral caudal extent of both hippocampi, subventricular zone progenitors that populate olfactory bulb and small numbers of non-neuronal cells throughout the brain (Pun et al., 2012). FB-*Pten* KOs produce widespread *Pten* loss among excitatory neurons in cortex and hippocampus with minimal involvement of other cell types. *Pten* loss in astrocytes has been shown to drive angiogenesis (Xiao et al., 2005), so relative inclusion of this cell type could be a key variable. Notably, however, cortical excitatory neurons were also targeted in f-*Tsc2* KOs, but with a more restricted distribution relative to the widespread targeting in the FB-*Pten* Kos, so at least in the f-*Tsc2* KO model, mTOR hyperactivation in cortical neurons appears to be sufficient to drive hypervascularization.

The developmental timing and duration of gene deletion could also modulate angiogenic responses to mTOR hyperactivation. *Pten* deletion in DGC-*Pten* KOs occurs among neural progenitor cells following tamoxifen administration to two-week old mice, while *Pten* deletion in FB-*Pten* KOs is delayed until a few weeks after birth, when expression of the CamkII promoter is robust (Tsien et al., 1996). By contrast, Emx1-Cre-driven and in utero electroporation approaches with *Tsc1* and *Rheb* both target immature cortical neurons, so gene deletion will occur earlier in development in these models (Zhang et al., 2019). The f-*Tsc2* KO model, however, also uses a later-acting CamkII promoter to drive cre-recombination, denying an obvious developmental explanation for the difference with the FB-*Pten* Kos, although the many other difference between models could be important (surgery, viral exposure, etc.). Possible technical differences between our *Pten* and *Tsc2* models are the use of DyLight 649-lectin to label the vessels in the *Pten* models and the within-animal analysis approach used in the *Tsc2* model. The lectin provides superior signal-to-noise than CD31 immunostaining, but has to be given *in vivo* and could not be used on existing fixed tissue from f-*Tsc2* KOs. On the other hand, the within-animal comparison approach used for the f-*Tsc2* KOs cannot be conducted in the *Pten* KOs, where both hemispheres were affected. Within-animal approaches can be statistically more powerful by controlling for interanimal variability, and thus might detect more subtle changes. Finally, we suggest that the duration of mTOR hyperactivation would not appear to be a driving factor for differing results, as prior work with *Tsc1* and *Rheb* found hypervascularization by P14 (Zhang et al., 2019), so animals in the present study harbored *Pten* KO cells for longer (albeit at later ages). Nonetheless, additional studies controlling for cell type, deletion extent and developmental timing with comparable techniques will be needed to establish whether the absence of hypervascularization in *Pten* KOs, and its presence in *Tsc1/Tsc2* KOs, reflects distinct gene function effects or other factors.

### Blood-brain barrier leakage

Failure of the blood-brain barrier is hypothesized to contribute to some forms of epilepsy (Dadas and Janigro, 2019). Introduction of serum proteins into the brain can directly evoke seizures and seizures can open the blood-brain barrier, creating the potential for an epileptogenic negative feedback loop (Gorter et al., 2019). Moreover, the mTOR antagonist rapamycin regulates vascular remodeling and blood-brain barrier leakage after status epilepticus (van Vliet et al., 2012, 2016a, b), indicating that a physiological function of the mTOR pathway (i.e., in the absence of any mutations) is to regulate the blood-brain barrier. This adds an additional level of complexity to understanding mTOR mutants, as mutations might drive initial pathology, such as seizures, and alter vascular remodeling responses to those seizures.

Systemic infusion with brain impermeant fluorescent tracers did not reveal significant evidence of leakage in DGC-*Pten* KO mice. Data with AF488-BSA is shown (Fig. 4), but pilot experiments with Evans Blue, various size dextran molecules and tomato lectin also showed no evidence of leakage (data not shown). By contrast, clear leakage was evident following pilocarpine-induced status epilepticus, an established cause of barrier failure (Ndode-Ekane et al., 2010; Bankstahl et al., 2018; Mendes et al., 2019). The findings suggest that ongoing and persistent blood-brain barrier failure is not a major contributor to seizure incidence in DGC-*Pten* KOs. We cannot exclude the possibility, however, that the leakage occurs only transiently. For example, immediately after seizure events or during specific developmental time windows. Notably, enhanced vessel growth, to accommodate the larger dentate gyri, was observed, and as new vessels have immature tight junctions and are thus more prone to leakage (Rust, 2020), it is possible that KO animals might exhibit leakage at earlier time points when vessel growth was maximal.

### Implications for mTORopathies

The present findings suggest that hypervascularization is not a universal consequence of mTOR pathway mutations, even when associated with gross hypertrophy of brain structures. Additional studies of clinical samples are needed to determine whether, and under what conditions, hypervascularization is prominent. Hypervascularization is a feature of tuberous sclerosis complex lesions (Laviv et al., 2018), and vascular abnormalities are evident in a variety of other disorders caused by mTOR pathway mutations (Hashizume et al., 2004; Martinez-Lopez et al., 2019). In addition to the genetic diversity of mTOR activating mutations, human conditions also exhibit extensive cellular diversity. Human disease-causing mutations can be germline, mosaic or a combination of both in the case of two-hit lesions in tuberous sclerosis complex (Martin et al., 2017; Reyna-Fabián et al., 2020). Affected cell types, therefore, could be a key determinant in whether vascular abnormalities develop. As shown in the present study, neuronal Pten loss can regulate VegfA protein levels, which could induce vascular changes and sustain neuronal hypertrophy. Studies in the cancer literature, however, also show that Pten can act on endothelial cells and astrocytes to drive angiogenesis (Gomez-Manzano et al., 2003; Su et al., 2003; Bhattacharya et al., 2013). As vascular pathology could be a key component of neurologic deficits associated with mTORopathies, understanding the variables driving vascular pathogenesis remains an important area of research.

## Synthesis

Reviewing Editor: William Stacey, University of Michigan Decisions are customarily a result of the Reviewing Editor and the peer reviewers coming together and discussing their recommendations until a consensus is reached. When revisions are invited, a fact-based synthesis statement explaining their decision and outlining what is needed to prepare a revision will be listed below. The following reviewer(s) agreed to reveal their identity: Matthew Weston, Bryan Luikart.

We agreed that these experiments demonstrated that, in these models, although the vasculature grows to support the increased dentate size that is evident at 10 wks of age, density is preserved and there is no BBB leakage evidence. Earlier timepoints show no change. Corroborating results are observed in the CaMKIIa model, although only one time point is tested due to constraints of the model. The findings are somewhat unexpected given the prior work on TSC and RHEB models.

In general there were only minor concerns. However, one question is whether the impact of this paper could be improved with some additional data. We at eNeuro are averse to asking authors for additional experiments, so if the data are not readily available this is not required. However, the following comment explains one area in which we think this paper could be greatly improved, if perhaps this analysis could be run from the prior experiments:

1. The results leave several open questions, which are addressed by the authors in the discussion but not experimentally. These include, whether other mTOR gene deletions (e.g. Tsc1) have fundamentally different effects, or whether other cell types (e.g. glia, vascular endothelial cells), or time points would cause more dramatic vascular reorganization. Performing these experiments is probably too much to ask for this paper, as they would take several months to a year to complete. The paper does not contain a lot of data, however, so adding some experiments would improve the overall impact of the paper. Perhaps there are additional protein targets involved in vascular remodeling that could be assayed by either Western or immunostaining.

Minor points:

2. “512 μm/pixel” resolution in the section describing the AF488-BSA experiments is likely a typo/mistake.

3. It is good that you caught the germline recombination events in the CaMKIIa line, but what is the rationale for keeping these animals in the study, if that is what is meant by “retained in the study.”

4. It would be good to see the pixel values for AF488-BSA fluorescence after Status next to the other two groups in Figure 4.

5. Please add some references that use BSA to assess BBB permeability.

6. Figure legend, Figure 4. (hippocampi/mouse) should be (sections/mouse)

7. In the introduction “Finally, the mTOR pathway regulates genes ....” Can the authors state how these genes have been shown to be regulated. IE is it an effect on translation or transcription? In what cell type has it been shown?

8. In the results, “A correlation analysis of Pten and Vegf protein levels....” Our feeling is that you simply do not have the “n” to drive such a correlation analysis, so would suggest removing this so as not to draw incorrect interpretation of the negative data.

9. In the discussion “The present findings are consistent with the interpretation that different mTOR-pathway genes have distinct effects on angiogenesis” We don’t think that this is the only plausible interpretation. Differences in the cell types that are genetically manipulated via IUE vs the transgenic strategy in the present study is an equally plausible explanation. Later in the paper, cell type specificity and timing are discussed. I would suggest changing the above verbiage indicating something like “a plausible mechanism for differences between studies in that different mTOR-pathway genes...”

10. There is not a citation or methods on how the PTEN IHC was performed in figure 1.

11. In figure 4 can the data for the wild-type, Pten KO, and the pilocarpine-induced SE positive control all be quantitated and presented in 4B.
